# Root Canal Stripping: Malpractice or Common Procedural Accident—An Ethical Dilemma in Endodontics

**DOI:** 10.1155/2016/4841090

**Published:** 2016-09-08

**Authors:** Ionela Elisabeta Ciobanu, Darian Rusu, Stefan-Ioan Stratul, Andreea Cristina Didilescu, Corina Marilena Cristache

**Affiliations:** ^1^Department of Orthodontics and Paedodontics, Faculty of Dental Medicine, University of Medicine and Pharmacy, Craiova, Romania; ^2^Department of Periodontology, Faculty of Dental Medicine, Victor Babes University of Medicine and Pharmacy, Timisoara, Romania; ^3^Division of Embryology, Faculty of Dental Medicine, “Carol Davila” University of Medicine and Pharmacy, Bucharest, Romania; ^4^Faculty of Midwifery and Medical Assisting, “Carol Davila” University of Medicine and Pharmacy, Bucharest, Romania

## Abstract

Root canal stripping is defined as an oblong, vertical perforation that appears especially in the middle section of curved root canals during endodontic treatments with nickel-titanium (Ni-Ti) instruments. Its occurrence may drastically affect the outcome of the treatment, transforming a common otherwise efficient endodontic procedure into a complication such as tooth extraction. In order to discuss the ethical and legal consequences, two cases of dental strip perforations are herewith presented. Due to the existence of risk factors for dental strip perforation, experience of the clinician and the use of magnification and modern imagistic methods (CBCT) may avoid or reduce the frequency of this type of accidents. Under correct working circumstances, dental stripping should not be regarded as a malpractice but as a procedural accident. However, the patient must always be informed, before and during the endodontic procedure, about the event and the possible complications that may occur.

## 1. Introduction

Endodontic stripping is an oblong, vertical perforation that occurs especially in the middle section of a curved root canal, caused by excessive instrumentation of the internal wall [1] during the removal of the organic material from the endodontic space and the tridimensional shaping of the canal by a progressive conical preparation. Stripping refers to a thinning of a curved wall followed, eventually, by its perforation. Ultimately, the discrete perforation and the subsequent extrusion of filling materials (sealer, gutta-percha, etc.) may lead to a lesion of endodontic origin and to progressive bone destruction. In some cases, the stripping occurs on a canal wall already thinned by a preexistent external root resorption, caused by an inflammatory lesion. However, perforations can also be caused by ledges, due to the inability to maintain the original canal morphology [2, 3]. The lateral lesion resulting from a stripping perforation is difficult to treat with conventional surgical approaches, mostly because of the restricted access. Directly linked to the loss of radicular dentin in the coronal two-thirds of the canal, the typical perforation by stripping also predisposes the root to premature fractures, which may lead to complications, such as tooth extraction [4]. If noted before the root canal filling procedure and if directly accessible under magnification, the practitioner can attempt to seal the stripping perforation with Mineral Trioxide Aggregate (MTA, Dentsply-Maillefer, Tulsa, USA), in an expensive and extremely technique-sensitive procedure [5], not accessible to all dental care practitioners. MTA has been recommended by Torabinejad et al. [6] as repair material for root perforations due to its better sealing ability than other materials. MTA has a high pH value (12.5), antimicrobial properties, and excellent biocompatibility, promoting the growth of cementum and bone formation, leading to the regeneration of the periodontal ligament around the site of injury [7]. Several authors [8–10] found, in human osteoblast model, that MTA stimulated the upregulation of cytokines, such as interleukin- (IL-) 1*α*, IL-1*β*, and IL-6, involved in bone turnover. All these properties make MTA a material of choice for repairing root perforations. In a reduced number of cases, when the perforation of the outer root wall is minute and the amount of extruded root canal filling material is negligible, the tooth can behave like it does after a usual postfilling extrusion, and, after a couple of days of tenderness to percussion, tooth remains indefinitely symptom-free. In all the situations, the costs and stress for the patient could become significant, raising an ethical dilemma: Is root canal stripping considered a malpractice or a procedural accident? What is the correct attitude that should be undertaken by the health care provider to minimize the potential harm or to avoid placing a patient at an unreasonable risk of harm?

In order to evaluate the ethical and legal aspects related to root stripping perforation resulting from an, otherwise correct, mechanical rotary preparation, two case reports are presented hereunder.

## 2. Case Report 1

**Figure 1:**
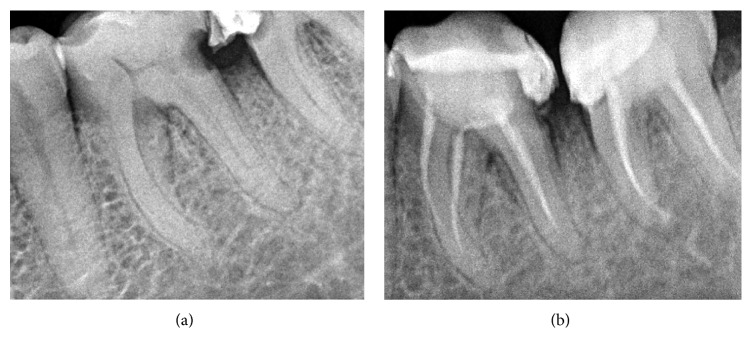
(a) Preoperative radiograph of the left mandibular first molar showing a deep carious lesion on the distal aspect of the crown. (b) Postoperative radiograph of the left mandibular first molar showing an incomplete and incorrect root canal filling, associated with changes of the initial curvature of the mesiobuccal canal.

A 67-year-old male patient was referred to a private dental practice, complaining of acute, pulsating pain in the mandibular left first molar (#36). In the case history, the patient reported episodes of acute pain, exacerbated during the night and temporarily alleviated with common painkillers. Pain intensifications were also diminished by rinsing with cold liquids in the affected molar region. The clinical oral examination revealed a deep carious lesion on the distal aspect of the crown, close to the cementum-enamel junction, and also a mesial decay lesion was observed at #37. Decayed dentin was present throughout the entire cavity, including the axial wall of both molars. For the first molar (#36), removing the decayed dentin led to an opening in the axial wall, with the occurrence of a puss droplet, followed by an abundant hemorrhage. The pain gradually subsided later and the tooth remained tender to percussion. Acute purulent pulpitis was established as positive diagnosis. In addition, a retroalveolar X-ray was performed (Figure 1(a)).

The root canal treatment was conducted in two appointments for both molars. In the first session, under local anesthesia, pulp extirpation was performed under 2.5% Sodium Hypochlorite irrigation, and a sterile cotton pellet was left in the pulp chamber. The cavity was sealed with temporary filling. In the second session, chemomechanical root shaping was performed using the manual nickel-titanium (Ni-Ti) ProTaper system according to the technique indicated by manufacturer (Dentsply-Maillefer, Ballaigues, Switzerland), in association with Sodium Hypochlorite and Ethylene Diamine Tetraacetic Acid (EDTA) irrigations. The root canals were dried using paper points. At this time, a discrete blood stain on the paper point between the cervical and middle thirds of the mesial root of tooth #36 was noted. Root canals were sealed with ProTaper gutta-percha and sealing cement. In the same session, the filling of the crown cavity with composite material was performed. Postsurgical retroalveolar X-ray performed showed an incomplete and incorrect root canal filling, associated with changes of the initial curvature of the canal (Figure 1(b)) for tooth #36 and a correct endodontic treatment for tooth #37. A light tenderness to percussion was recorded at the first molar, while the second molar was asymptomatic. Despite the fact that he was informed about all risks and treatment options (including tooth extraction or #36 tooth retreatment and MTA sealing under microscope), the patient's final decision was to keep the tooth under observation without any additional treatment.

## 3. Case Report 2

A 52-year-old male patient was referred to a private dental practice for endodontic advice and treatment. The patient was complaining about discomfort in the mandibular right molar region and the case history revealed spontaneous acute pain exacerbated by cold drinks, occurring a couple of months before. Intake of common painkillers helped to alleviate the pain, which disappeared later. Intraoral clinical examination revealed two metallic crowns on the right first and second molars. In addition, a retroalveolar X-ray of the tooth was performed showing a canal wall already thinned by a preexistent external distal root resorption, caused by an inflammatory lesion. After tooth opening through the metal crown, decayed dentin was noticed, including the axial wall. The opening of the pulp chamber occurred while removing the decayed dentin. Pain was not present at the opening time. The root canal probing revealed sensitivity and hemorrhage, while the tenderness to percussion test was negative. The positive diagnosis of chronic pulpitis was set. The root canal treatment was conducted in two sessions. In the first session, under local infiltration anesthesia, pulp extirpation was performed, irrigations with 2.5% Sodium Hypochlorite were carried out, a cotton pellet with a mixture of antibiotic and steroidal anti-inflammatory (Ledermix, Riemser Pharma GmbH, Greifswald, Germany) was introduced in the pulp chamber, and the cavity was sealed with temporary filling.

**Figure 2:**
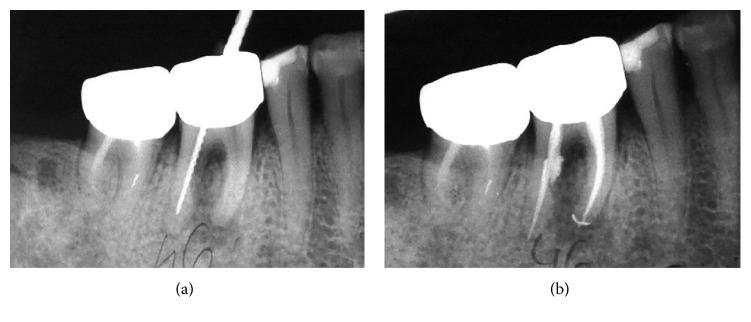
(a) The endodontic lesion extended from the apex of the mesial root of tooth 4.6. towards the furcation dome, causing an external resorption of the mesial aspect of the distal root. (b) The regular shaping with nickel-titanium (Ni-Ti) instruments of the distal canal resulted in stripping with extrusion of the filling material, which became obvious only on postoperative radiographic examination.

During the second appointment, a rigorous chemomechanical root shaping was performed. Manual K-files, size 15–40, were used just to avoid a potentially dangerous enlargement of the distal and mesial canals. However, because of the initial curvature of the canal, a relocation of the canal orifice to mesial became obvious even during the mechanical preparation and a retroalveolar X-ray with manual K-file was performed. Irrigations with 2.5% Sodium Hypochlorite and EDTA were also performed. While drying the canal, blood impregnation of the paper point was observed at the middle third level of the distal root. In the same appointment, the root canals were filled using the lateral cold condensation technique. Endomethasone (Septodont, Saint-Maur-des-Fosses, France) was used as sealer. Temporary coronal filling was performed. The postoperative retroalveolar X-ray showed a slight overfilling of the distal root canal, a thinned distal wall of the mesial root, and a nonhomogenous radioopaque mass protruding in the periodontal space at the mid-level of the root canal (Figure 2(b)). Patient was informed about the event and advised about the treatment options, including tooth extraction or using MTA applied under a surgical microscope. The second option was preferred by the patient. Due to incorrect root canal filling of tooth #47 as well, both molars were retreated after referral to an endodontist specialist.

## 4. Discussion

In order to analyze the ethical aspect of root canal stripping in both cases, the four-topic method (medical indications, patient preferences, quality of life, and contextual features) described by Jonsen et al. [11] were used for focusing on specific aspects and for connecting the circumstances of the cases to their underlying ethical principles.

The topic of medical indications for both cases addressed treating the root canal by the removal of the organic material from the endodontic space and the tridimensional shaping by a progressive conical preparation, with a minimum diameter at the apical constriction level, in order to maintain its initial shape [12]. Unfortunately, these objectives are often difficult to achieve in many cases, due to anatomical irregularities of the canal, accentuated curvatures, and/or presence of lateral and accessory canals [13, 14]. Maintenance of the initial shape of the canal becomes in such circumstances difficult.

Mandibular molars have been reported to be more susceptible to strip perforations due to their root anatomy [15, 16], directing to the fourth topic addressed, circumstances of the cases.

Most of the root canals have a certain degree of curvature, while endodontic files, both manual and rotary, are straight and tend to straighten in curved root canals. Even precurved steel files tend to straighten once placed into the root canal due to the bending moment of the instruments. In the second case report, even stainless steel K-files may be considered as risk factors for stripping when excessively shaping the root canal.

Rotary Ni-Ti root canal files represent a widely used alternative to stainless steel instruments in endodontics [17]. Flexibility and resistance to torsion fracture of this alloy made it possible to improve the design features of endodontic instruments, reducing errors and facilitating a better preservation of the root canal anatomy [18]. However, the large taper of these files predisposes to stripping, when used in thin, curved roots. In the first case presented, Ni-Ti files with progressive taper were used, which would explain the stripping occurring in the cervical portion of the canal in case of a thin wall and a thin pulpal floor at this level. Obviously, in the two cases presented above, the root canal morphology was not sufficiently analyzed, the endodontic treatment planning was inadequate, and the relocation of the canal was not kept under control. Moreover, thin roots with severe curvatures may require a Cone Beam Computed Tomography (CBCT) evaluation prior to and during the root canal rotary shaping, in order to determine the remaining distance to the concave outer wall or the presence of adjacent erosive inflammatory lesions.

In both reported cases, the molar roots presented an anatomy that predisposed to the risk of stripping. In the first case, the mesial root had curvatures in more than one plane, and in the second case the mesial wall of the distal root had a reduced thickness, requiring a shaping technique that would have prevented the excessive thinning of the mesial wall.

Regarding patients preferences, case management included informed consent about what happened and advice about the treatment options. Referral to a specialist was decided to be in the best interest of the second patient. The first patient preferred follow-up and tooth extraction if needed. Treatment and prognosis differed from those of a lateral root perforation because of the size, oval shape, and thin edges of the stripping [19]. If extrusions are minute, it is recommended to inform the patient and to keep the tooth under observation with regular X-ray checks to detect any development of an endodontic lesion. If the latter becomes apparent and seems to evolve, endodontic microsurgery with placement of MTA is recommended as a first choice.

When analyzing quality of life, significant improvement was noticed in both cases, despite the reserved long-term prognosis.

Regarding malpractice versus procedural accident, medical malpractice is the negligence arising out of the doctor-patient relationship, whereas negligence is the unreasonable act or omission by a provider that results in patient harm [20]. According to Seidberg [21], some of the common elements of malpractice and negligence are failure to meet the standard of care, practice beyond the scope of license, performing procedures the doctors are not competent to do, delegating to a nonqualified person, using materials not meeting standards, insurance fraud, failure to diagnose, and failure to refer.

The specialty of endodontics, like all other dental specialties, is guided by each country's National Code of Ethics issuing guidelines (templates and not mandatory) as national standard of care. The accepted definition for standard of care is “that reasonable care and diligence ordinarily exercised by similar members of the profession in similar cases in like conditions given due regard for the state of the art” [20]. National standards have replaced local rules because of the ease of obtaining continuing education from local or national educational programmes or from the dental literature. As reported by Seidberg [22], the standards are usually set by the expert witnesses by citing a specialty organization's guidelines as a basis for their evidence for the specific case for which they are testifying. The mandatory ethical concepts of an adequate standard of care are to recommend the best therapy while minimizing potential harm and to avoid placing a patient at an unreasonable risk of harm that cannot be disputed in court by an opposing expert witness. Evidence provided may include elements of location, availability of facilities, specialization or general practice, proximity of specialists, and special facilities as well as other relevant considerations. General dentists are usually held to the same standard of care as those of specialists when performing that particular phase of dentistry [22].

According to the Law on Healthcare Reform in Romania (Law Number 95/2006) [23], as a member of the European Union (EU), malpractice is defined as being the professional error committed during the medical or medicopharmaceutical act, harmful for the patient, involving civil liability of healthcare professionals and providers of medical, healthcare, and pharmaceutical products and services.

In our country, the recently introduced specialty of endodontics is guided by the Code of Ethics of the National Romanian College of Dentists [23]. The code regulates rather the abovementioned doctor-patient relationship in case of an alleged malpractice case, while the ascertainment of a malpractice is usually done by the Malpractice Commission of the Districtual Health Authority, for judiciary purposes. Standard of care in endodontic treatment in our country, for example, does not include the following as being mandatory for endodontic treatment: preoperative/intraoperative CBCT and microscope endodontic surgery. This is in agreement with quality guidelines for endodontic treatment of the European Society of Endodontology [24]. According to these circumstances, perforation by stripping should be considered an accident when the accentuated root curvature is associated with very thin root walls. Unfortunately, in most of the cases, this is the result of the lack of care or lack of experience of the dentist. More specific norms for defining standard of care are needed.

## 5. Conclusions

Despite the existence of risk factors for dental strip perforation, such as root canal anatomy and morphology as well as elastic (memory-shape) properties of modern endodontic instruments, the experience of the dental clinician is important in order to avoid or reduce the frequency of this type of accidents. Like in so many other complex situations in dentistry, the practitioner may encounter unwanted and unforeseen circumstances during root canal therapy that may affect the prognosis. Therefore, under correct working circumstances, dental stripping should not be considered a malpractice event but a procedural accident. Whenever possible, magnification and modern imagistic methods such as CBCT should be used. However, in similar cases, the patient must be informed about possible complications that may appear before and after the treatment if stripping occurs.
